# Supplementary material for: The Human TOR Signaling Regulator Is the Key Indicator of Liver Cancer Patients’ Overall Survival: TIPRL/LC3/CD133/CD44 as Potential Biomarkers for Early Liver Cancers
Source: Cancers (Basel). 2021 Jun 11;13(12):2925. doi: 10.3390/cancers13122925 (PMC8230774; doi:10.3390/cancers13122925)
Supplement: Supplementary file 1 [file cancers-13-02925-s001.zip › cancers-1253676-supplementary_0611.pdf]

cancers

MDPI

The Human TOR Signaling Regulator is the Key Indicator of Liver Cancer Patients' Overall Survival: TIPRL/LC3/CD133/CD44 as Potential Biomarkers for Early Liver Cancers

Soo Young Jun, Hyang Ran Yoon, Ji-Yong Yoon, Su-Jin Jeon, Jeong-Ju Lee, Debasish Halder, Jin-Man Kim and Nam-Soon Kim

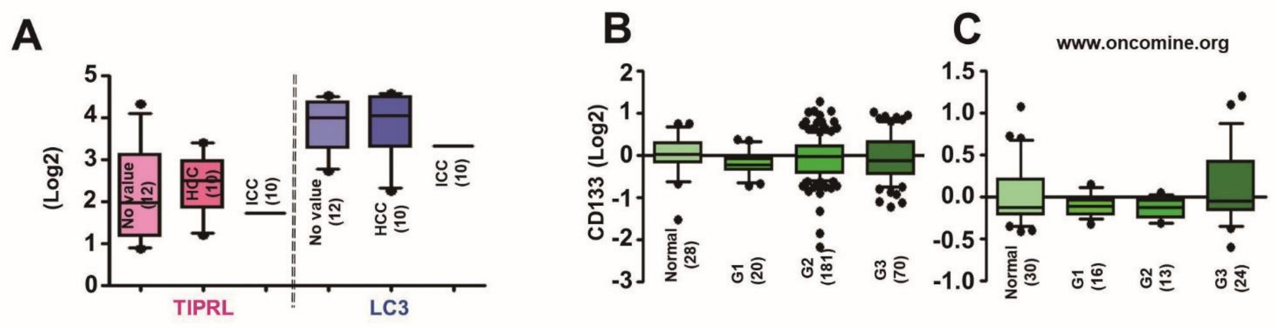

Figure S1. The expression level of the variables in HCCs and iCCA. (**A**) The levels of the variables in HCCs and iCCA were obtained from the public database (www.oncomine.org). The level of CD133 in each grade from our cohort (**B**) and the public database (**C**). (*n*), the number of the sample. No value, tissues cannot be categorized to any grade.

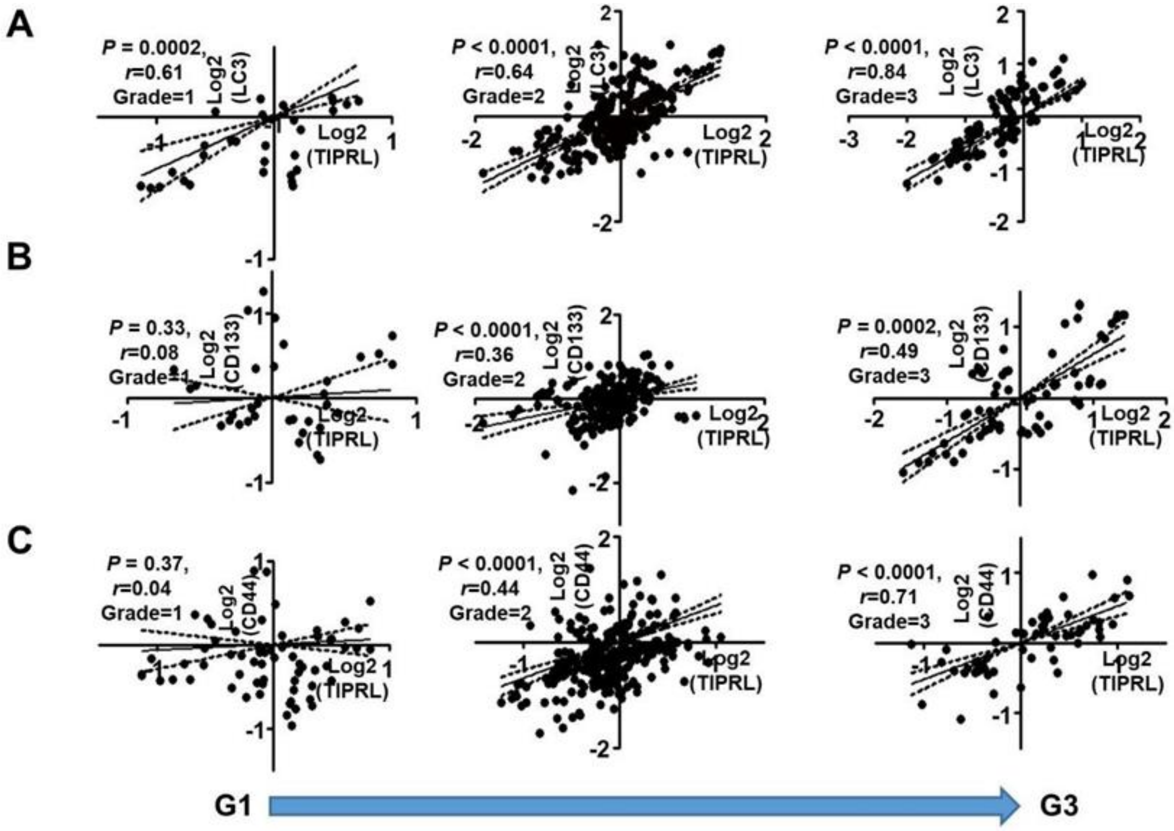

Figure S2. The significant association between TIPRL and LC3/CD133/CD44 in HCCs. HCCs were categorized into their cancer grades and then evaluated for the correlation with the variables according to cancer grades. x, TIPRL(Log2); y, LC3 (Log2, **A**), CD133 (Log2, **B**), CD44 (Log2, **C**), *P*-values, Spearman *r*, and Grade were represented. A single dot indicates one sample.

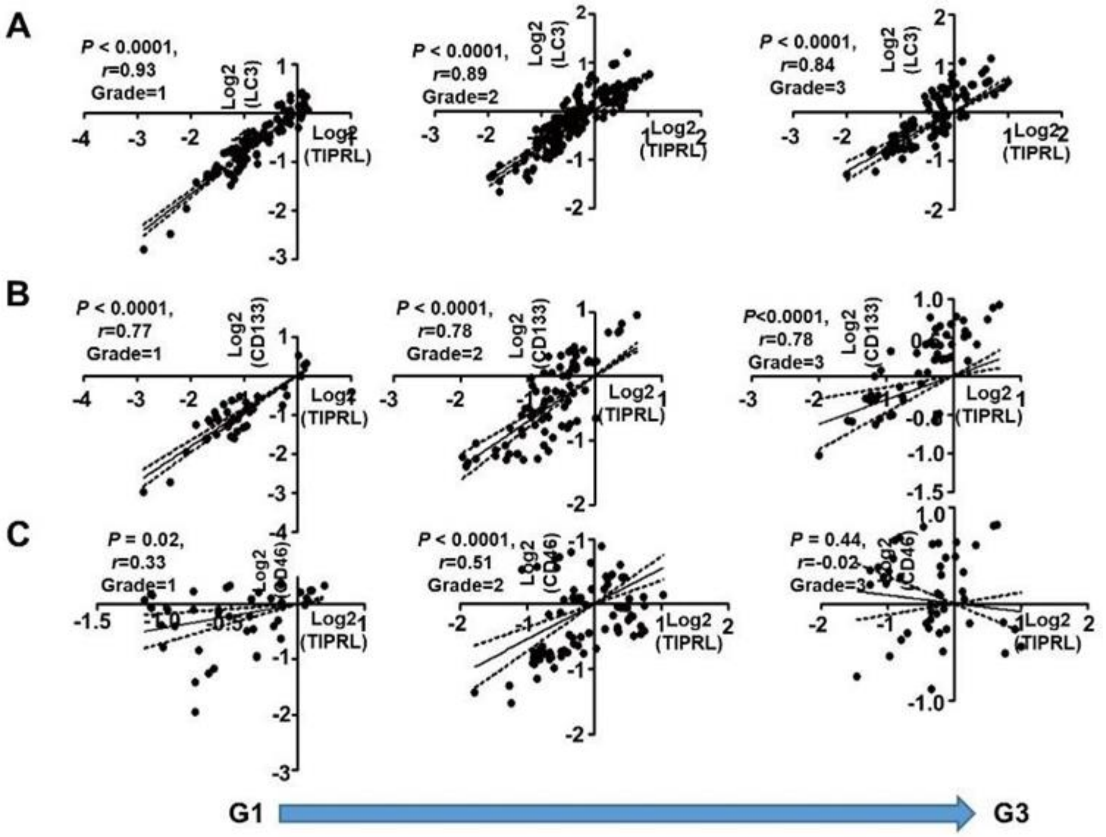

Figure S3. The significant grade-dependent increase in the association between TIPRL and LC3/CD133 in iCCA. iCCA were grouped into their cancer grades and then evaluated for the correlation with the variables according to cancer grades. x, TIPRL(Log2); y, LC3 (Log2, **A**), CD133 (Log2, **B**), CD46 (Log2, **C**), *P*-values, Spearman *r*, and Grade were represented. A single dot indicates one sample.

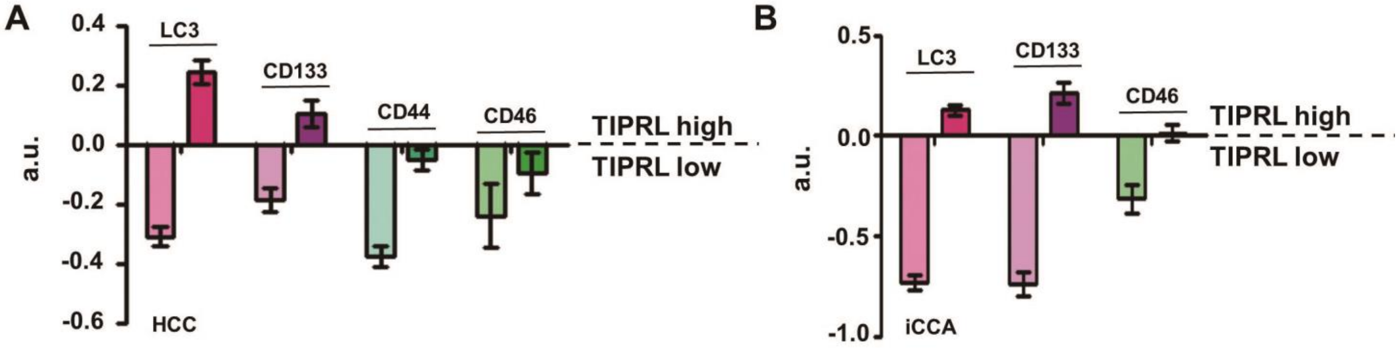

Figure S4. The significant relationship between TIPRL and LC3/CD133 in HCCs and iCCA. TIPRL level was subdivided into high and low expressed group, and then the level of the variables, LC3/CD133/CD44/CD46 (**A**), and LC3/CD133/CD46 (**B**) was evaluated in theTIPRL low and high expressed group. a.u. artificial unit and standard error.

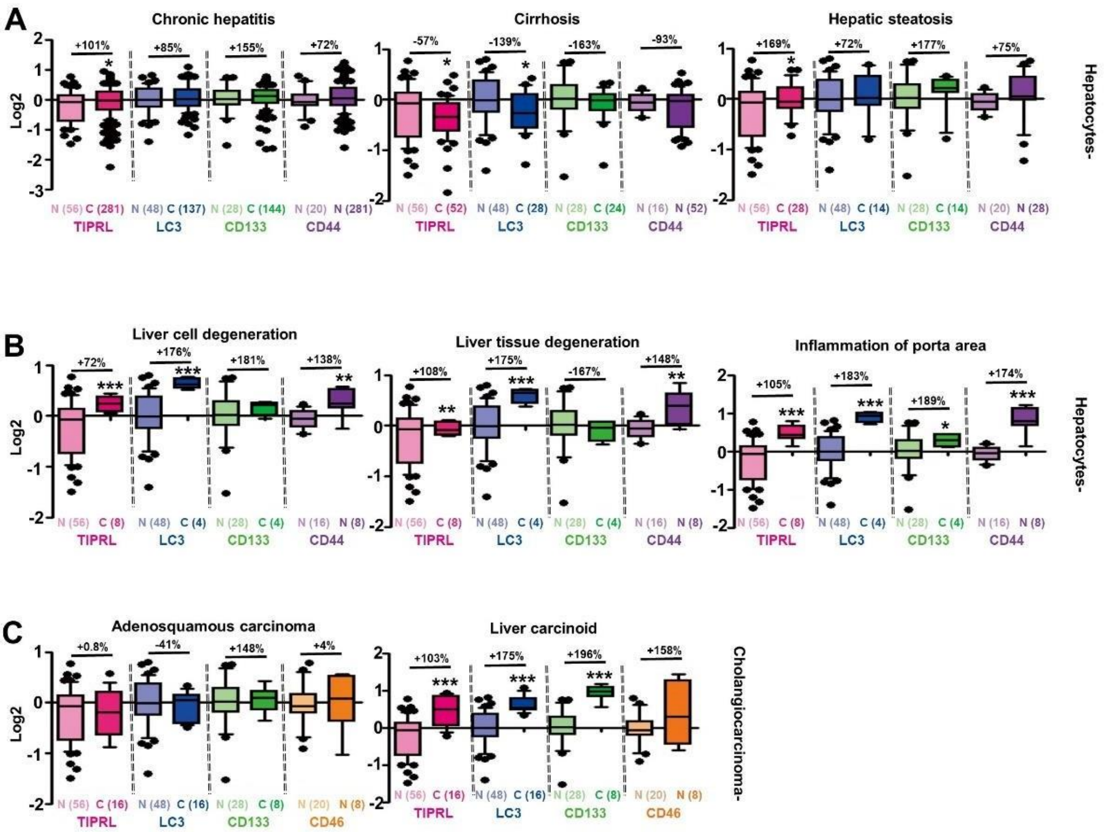

Figure S5. Differential expression of the variables in different liver disease tissues. The levels of the variables in different liver disease tissues (Table S2–3) were categorized into each group according to their disease name. Each set was grouped, given its origins, such as hepatocytes-originated (**A**, **B**) and cholangiocarcinoma-originated (**C**). * $p < 0.05$, ** $p < 0.01$, *** $p < 0.001$ were determined by a paired t-test, and % differences are noted. (n), the number of the sample.

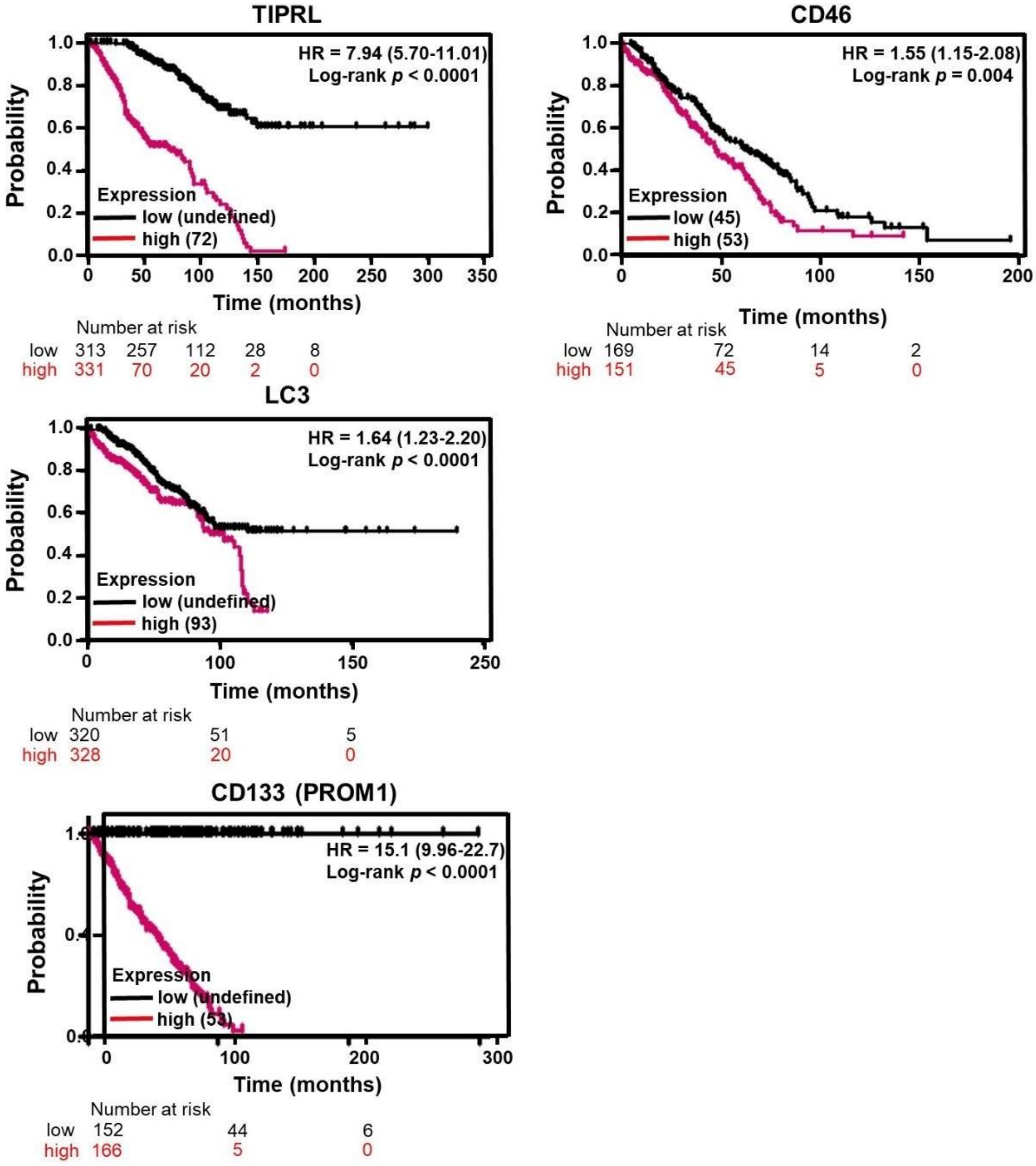

Figure S6. The survivability of liver disease patients in the validation set. The survival time of liver disease patients in the validation set was determined using the Kaplan Meier estimator. HR, (95%CI), Log-rank, and *p*-values arenoted.

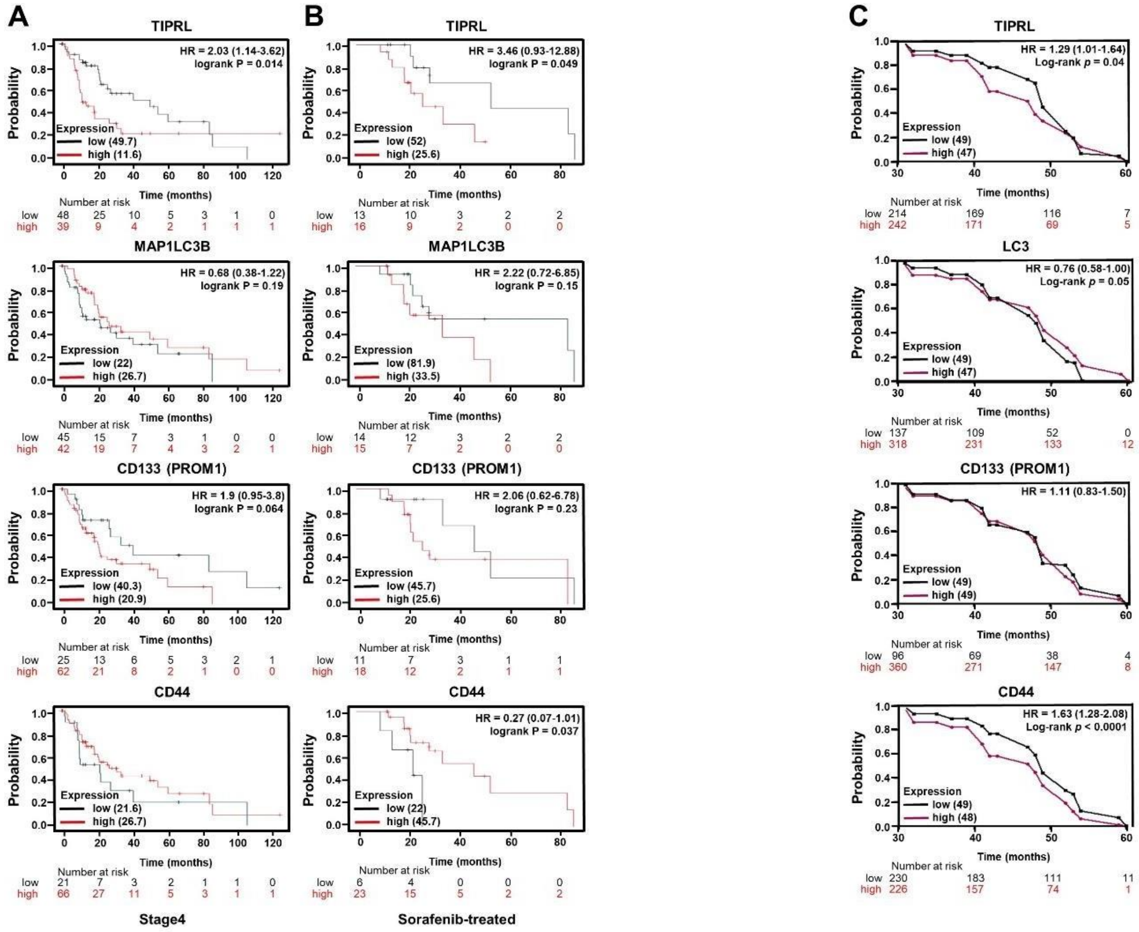

Figure S7. TIPRL has a prominent effect on liver cancer patients' survival. A public database (www.kmplot.com accessed on 12–28 January 2021) was used to investigate the relationship between the level of the variables and patients with stage 4 liver cancers (**A**) or soraf-enib-treated liver cancer patients (**B**). (**C**) The DSS of liver cancer patients in the training set was determined using the Kaplan Meier estimator. HR (95%CI), Log-rank, and *p*-values are noted.

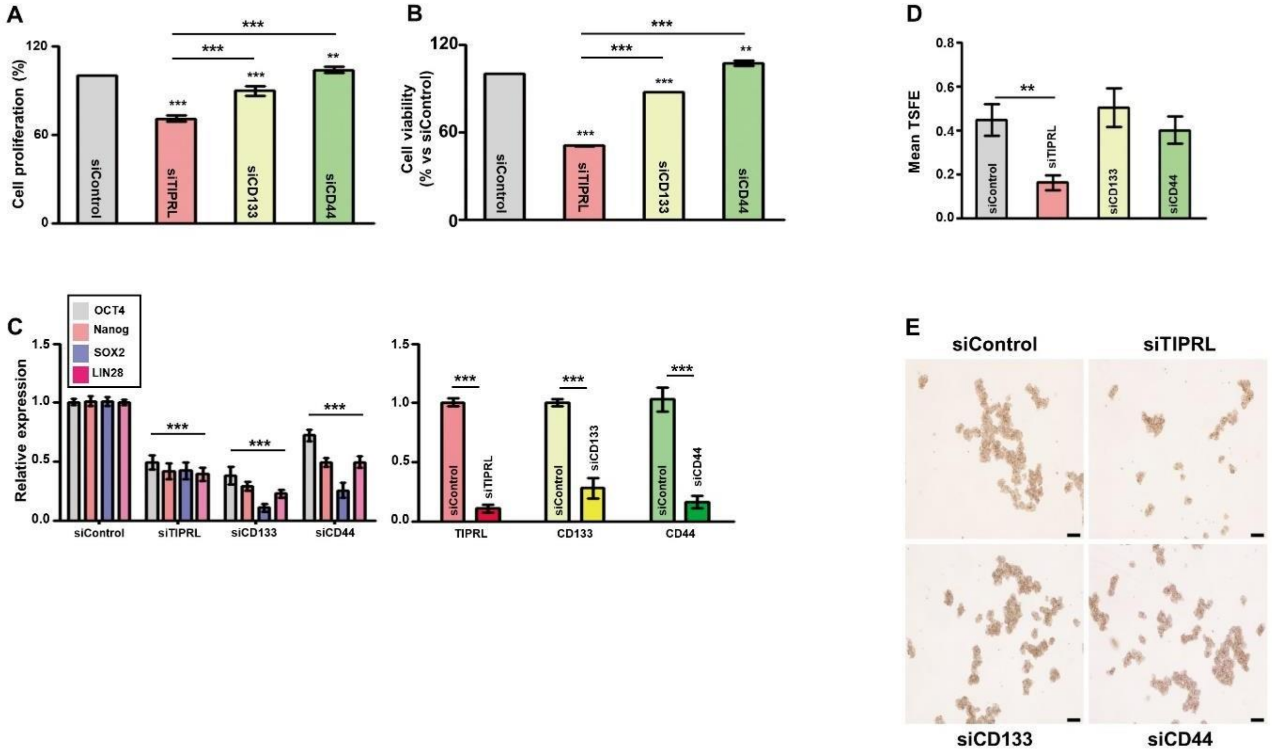

Figure S8. TIPRL is a crucial player in SNU1097, iCCA cell viability and stemness. SNU1097 cells were seeded in a 96-well plate (**A**) or anoikis plates (**B**–**E**) followed by transfected with the indicated siRNAs. For cell proliferation (**A**) and viability (**B**) assays, 48 hours after siRNA transfection, an MTT assay was performed. For quantification analysis of expression in stemness-related genes, we performed RT-qPCR using primers (C; Table S12). (**D**–**E**) We counted the numbers of spheroids after 72 hours siRNAs transfection. TSFE, tumorsphere formation efficiency: [(the number of tumorspheres formed/the initial number of cells seeded) × 100]. All experiments were independently repeated three times. ** $p < 0.01$, *** $p < 0.001$ by unpaired *t*-test.

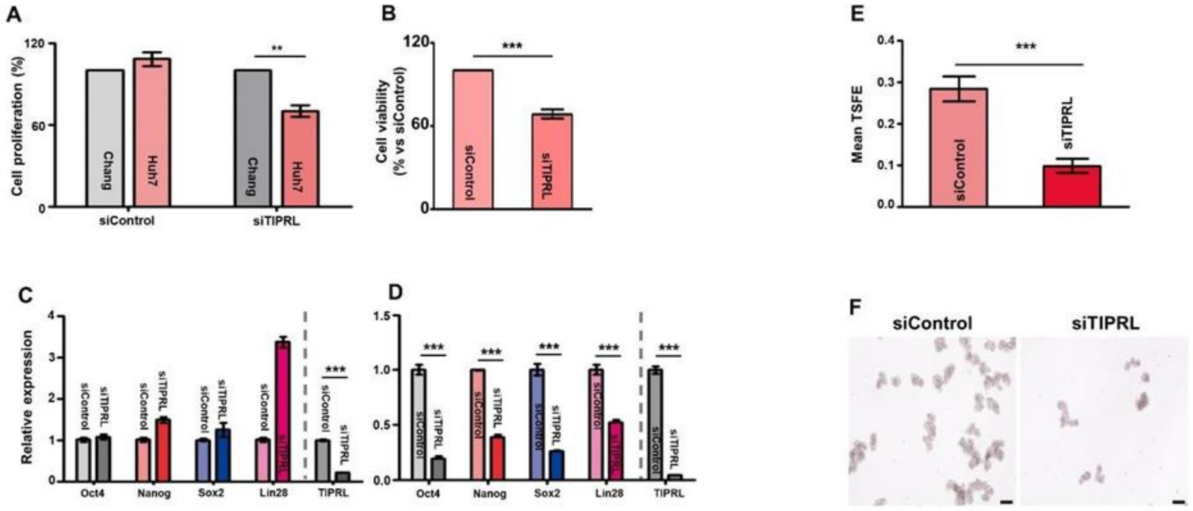

Figure S9. The critical role of TIPRL in huh7, HCC cell viability and stemness Cells were transfected with second siTIPRL (Table S12) were seeded in a 96-well plate (**A**) or anoikis plates (**B**–**F**). For cell proliferation (**A**) and viability (**B**) assays, 48 hours after transfection, an MTT assay was performed. For quantification analysis of expression in stemness- related genes, we performed RT-qPCR using primers (**C**–**D**; Table S12). (**E**–**F**) We counted the numbers of spheroids after 72 hours siRNAs transfection. TSFE, tumorsphere formation efficiency: [(the number of tumorspheres formed/the initial number of cells seeded) × 100]. All experiments were independently repeated three times. ** $p < 0.01$, *** $p < 0.001$ by unpaired *t*-test.

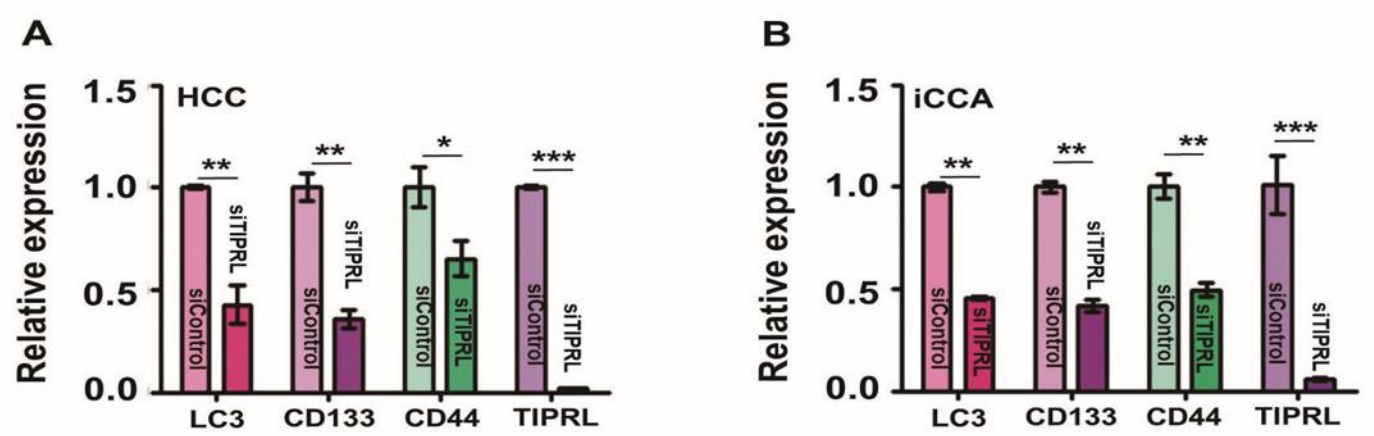

Figure S10. The depletion of TIPRL decreased significantly expression of LC3, CD133, and CD44. Huh7, HCC (**A**) and SNU-1097, iCCA (**B**) cells were transfected with siTIPRL (100 nM). 72 hrs after that, rt-qPCR analyses for the indicated primers (Table S12) were performed. $n = 3$. *$P < 0.05$, ** $P < 0.01$, *** $P < 0.001$ by two-way anova with bonferroni post hoc test. $n = 3$.

A

HCC/grade 1	Diagnosis					
	AUC (95% CI, %)			cut-off (95% CI, %)		
TIPRL	59.3%, (0.49-0.70), *p*= 0.09	Sensitivity	51.6 (38.7-64.3)	-0.25	Sensitivity	71.9 (59.2-82.4)
		Specificity	51.0 (36.6-65.3)		Specificity	45.1 (31.1-59.7)
LC3	53.7%, (0.41-0.66), *p* = 0.58	Sensitivity	54.8 (36.0-72.7)	0.15	Sensitivity	100 (88.8-100.0)
		Specificity	52.9 (38.5-67.1)		Specificity	23.5 (12.8-37.5)
CD133	61.4%, (0.49-0.74), *p* = 0.08	Sensitivity	48.5 (30.8-66.5)	-0.74	Sensitivity	100 (89.4-100)
		Specificity	51.0 (36.6-65.3)		Specificity	27.5 (15.9-41.7)
CD44	51.0%, (0.40-0.62), p=0.84	Sensitivity	59.7 (54.7-64.7)	-0.16	Sensitivity	56.4 (51.3-61.4)
		Specificity	54.6 (23.4-83.3)		Specificity	72.7 (39.0-94.0)
TIPRL/LC3/CD133	56.7%, (0.47-0.67), *p* = 0.16	Sensitivity	50.0 (41.0-59.0)	-0.70	Sensitivity	93.8 (88.1-97.3)
		Specificity	51.0 (36.6-65.3)		Specificity	29.4 (17.5-43.8)
TIPRL/LC3/CD133/CD44	51.5%, (0.43-0.60), p = 0.71	Sensitivity	54.0 (49.6-58.4)	-0.05	Sensitivity	63.0 (58.6-67.2)
		Specificity	53.2 (40.1-66.0)		Specificity	48.4 (35.5-61.4)

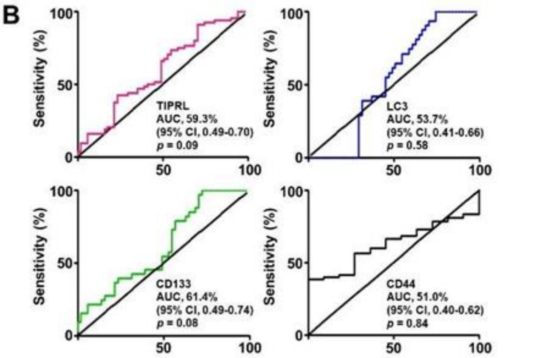

C

HCC/grade 1	Prognosis					
	AUC (95% CI, %)			cut-off (95% CI, %)		
TIPRL	76.5%, (0.68-0.85), *p* < 0.0001	Sensitivity	51.5 (39.0-63.8)	-0.01	Sensitivity	100.0 (94.7-100.0)
		Specificity	51.5 (39.6-63.8)		Specificity	51.5 (39.0-63.8)
LC3	81.1%, (0.74-0.88), *p* < 0.0001	Sensitivity	51.5 (39.0-63.8)	-0.004	Sensitivity	100.0 (94.7-100.0)
		Specificity	85.3 (74.6-92.7)		Specificity	36.8 (25.4-49.3)
CD133	75.3%, (0.67-0.83), *p* < 0.0001	Sensitivity	51.5 (39.0-63.8)	0.97	Sensitivity	51.5 (39.0-63.8)
		Specificity	77.9 (66.2-87.1)		Specificity	97.1 (89.8-99.6)
CD44	87.2%, (0.81-0.93), *p < 0.0001*	Sensitivity	51.5 (39.0-63.8)	-0.01	Sensitivity	100.0 (94.7-100.0)
		Specificity	73.5 (61.4-83.5)		Specificity	73.5 (61.4-83.5)
TIPRL/LC3/CD133	77.6%, (0.73-0.82), *p* < 0.0001	Sensitivity	51.5 (44.4-58.5)	-0.004	Sensitivity	100.0 (98.2-100.0)
		Specificity	71.6 (64.9-77.7)		Specificity	38.2 (31.5-45.3)
TIPRL/LC3/CD133/CD44	78.6%, (0.75-0.82), *p* < 0.0001	Sensitivity	51.5 (45.4-57.6)	-0.01	Sensitivity	100.0 (98.7-100.0)
		Specificity	70.2 (64.4-75.6)		Specificity	44.5 (38.5-50.6)

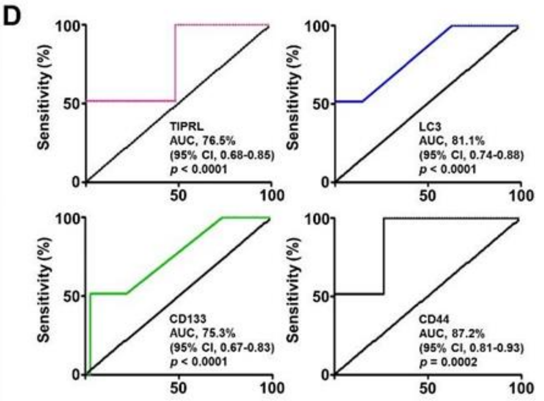

Figure S11. The prominent efficiency of TIPRL, LC3, CD133, and CD44 as the prognostic marker. ROC analysis was used to determine diagnostic (**A**–**B**) and prognostic (**C**–**D**) abilities of the variables in grade 1 HCCs. AUC, the area under the curve, (95% CI), and *p*-values are noted.

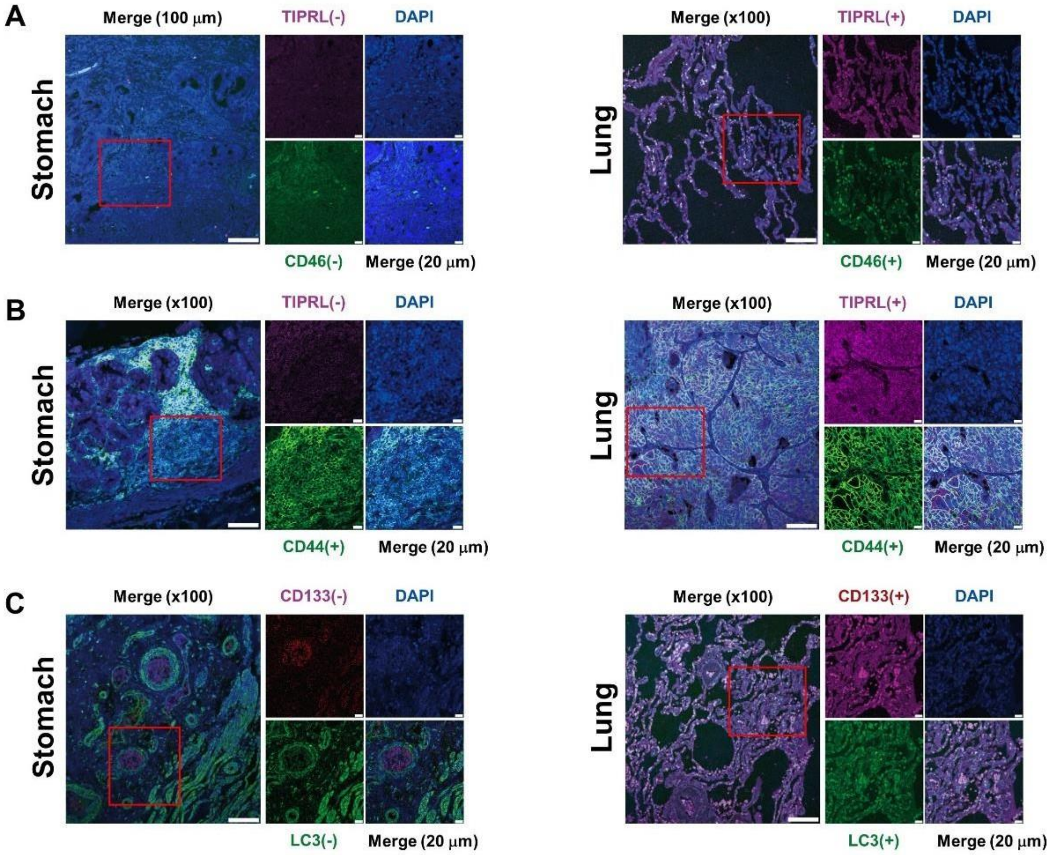

Figure S12. Demonstration of the antibody specificity using human stomach and lung tissues. Human stomach and lung tissues were stained with the indicated antibodies and then observed under confocal microscope. DAPI was used for staining nucleus, and scale bars, 20 and 100 μm. (−) and (+) indicate negative and positive staining, and the information were obtained from "The human protein atlas (https://v15.proteinatlas.org/cancer accessed on 1 June 2021).
